# Decreased expression of HLA-DR antigen-associated invariant chain mRNA predicts mortality after septic shock

**DOI:** 10.1186/cc11698

**Published:** 2012-11-14

**Authors:** M-A Cazalis, L Cavé, J Demaret, V Barbalat, E Cerrato, A Lepape, A Pachot, G Monneret, F Venet

**Affiliations:** 1 Joint Unit 'Sepsis', Hospices Civils de Lyon - bioMérieux, Hôpital Edouard Herriot, Lyon, France; 2 Immunology Laboratory, Hôpital Edouard Herriot, Lyon, France; 3 Intensive Care Units, Centre Hospitalier Lyon-Sud, Pierre Bénite, France

## Background

Septic syndromes remain the leading cause of mortality in ICUs. Patients rapidly develop immune dysfunctions of which intensity and duration have been linked with increased risk of secondary ICU-acquired infections and death. A decreased expression on circulating monocytes of human leukocyte-antigen DR (mHLA-DR) measured by flow cytometry has been shown to be a good marker of sepsis-induced immune dysfunctions [1] and to correlate with increased risk of death and ICU-acquired infections [2,3]. However, pre-analytical and analytical issues inherent to mHLA-DR measurement by flow cytometry limit the use of this marker in large multicentric clinical studies and on a routine basis. We investigated whether the whole blood mRNA expression of genes related to major histocompatibility class II (MHC class II) antigens could correlate with mHLA-DR protein expression measured by flow cytometry and predict mortality in septic shock patients.

## Methods

Ninety-three septic shock patients were included. PAXgene^® ^tubes were collected at days 3 to 4 after the onset of shock. The mRNA expression of five MHC class II-related genes (that is, CD74, HLA-DRA, HLA-DMB, HLA-DMA and CIITA) was measured by qRT-PCR. In parallel, ethylene diamine tetraacetic acid-anticoagulated blood samples were collected to measure mHLA-DR expression by flow cytometry.

## Results

**Figure 1:**
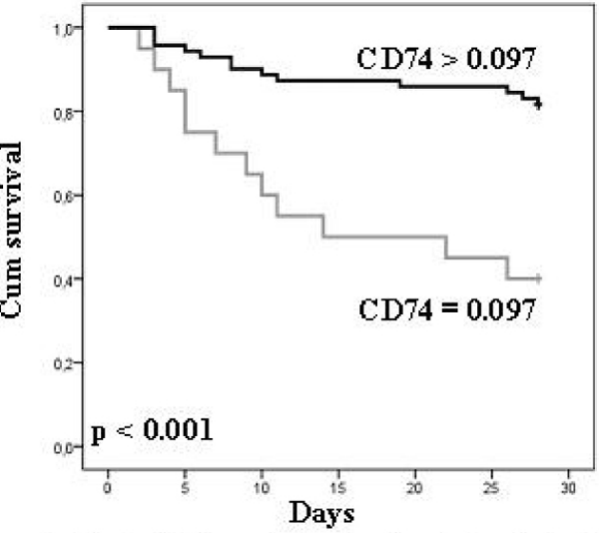
**Kaplan-Meier analysis of septic shock patients' 28-day survival after stratification on CD74 mRNA expression**. The threshold was chosen based on the Youden index calculated on the receiver operating characteristic curve. There is a significant difference between the two curves (log-rank test, *P *< 0.001; hazard ratio = 7.065, 95% CI = 2.56 to 19.48)

A significant correlation (*r *> 0.8) was found between the mRNA expression of MHC class II related genes. As observed for mHLA-DR, the mRNA levels were significantly decreased in nonsurvivors as compared with survivors. The best predictive value was obtained for the invariant chain (CD74) mRNA level (*P *= 0.003). This was confirmed by the receiver operating characteristic curve analysis (AUC = 0.7, 95% CI = 0.574 to 0.822; *P *= 0.003). Importantly, this remained significant after multivariate analysis including usual confounders (severity scores) and mHLA-DR values (*P *= 0.016). Survival curves showed that the decreased CD74 mRNA level was associated with increased mortality after septic shock (Figure 1).

## Conclusion

Decreased invariant chain mRNA expression significantly predicts 28-day mortality in septic shock patients. After validation in a larger multicentric study, this biomarker could become a robust predictor of mortality in septic patients. This is all the more because the availability in routine laboratories of molecular biology platforms will enable standardized and routine use of such biomarkers.
